# Preceptor characteristics and the socialization outcomes of new graduate nurses during a preceptorship programme

**DOI:** 10.1002/nop2.58

**Published:** 2016-06-29

**Authors:** Michelle Lalonde, Linda McGillis Hall

**Affiliations:** ^1^ School of NursingFaculty of Health SciencesUniversity of OttawaOttawaOntarioCanada; ^2^ Lawrence S. BloombergFaculty of NursingUniversity of TorontoTorontoOntarioCanada

**Keywords:** emotional intelligence, new graduate nurse, nurses, nursing, personality, preceptorship, socialization

## Abstract

**Aim:**

The purpose of this study was to explore the relationships between preceptor characteristics (emotional intelligence, personality and cognitive intelligence) and new graduate nurse socialization outcomes regarding turnover intent, job satisfaction, role conflict and ambiguity during a preceptorship programme. To date, no studies have explored these relationships.

**Design:**

A cross‐sectional and multi‐site design with purposeful sampling.

**Methods:**

Dyads of preceptors and new nurses were recruited at the end of their preceptorship programme. Pearson's correlational analysis was used to examine the relationships.

**Results:**

A sample of 41 preceptors and 44 new graduate nurses participated in this study, making 38 dyads with complete data. The preceptor personality traits of openness, conscientiousness and emotional stability were significantly related to new graduate nurses who reported greater turnover intent, job dissatisfaction, role conflict and ambiguity. No significant relationships were noted between preceptor EI and IQ and the outcome of new graduate nurses.

## 1 Introduction

A preceptorship programme is a formal teaching and learning method whereby a preceptor, or an experienced nurse and a new graduate nurse work together for a specified duration of time to assist novices in effectively adjusting to their new role, performing their tasks, gaining a basic level of knowledge and skills and socially adapting to the practice, profession and organization, while bridging the theory–practice gap (Canadian Nurses Association, 2004). One of the underlying assumptions of preceptorships is that a consistent relationship with one preceptor will assist in the socialization of the new nurse regarding the practice setting (Luhanga, Billay, Grundy, Myrick, & Yonge, 2010). This suggests that preceptors, acting as socializing agents, are an important component of preceptorship and the socialization of new graduates. Therefore, it is essential to consider and explore preceptor characteristics to gain a better understanding of what distinguishes a bad preceptor from a good one. One characteristic for consideration is the preceptor's emotional intelligence (EI). The literature suggests that this aspect may play an important role in workforce integration (Lopes, Salovey, & Straus, 2003; Sy, Tram, & O'Hara, 2006). Emotional intelligence is defined as ‘the ability to monitor one's own and others’ emotions, to discriminate among them and use the information to guide one's thinking and actions’ (Salovey & Mayer, 1990, p. 189). Research has demonstrated that the concept of EI shares variance with personality and cognitive intelligence (IQ) (Mayer, Salovey, & Caruso, 2004; Schulte, Ree, & Carretta, 2004) and is therefore important to consider when examining the influence of these two variables as well. To date, no literature has examined the effects of preceptor EI, personality and IQ on the socialization outcomes of new nurses during a preceptorship programme.

### 1.1 Background

Organizational socialization is defined as the ‘process by which an individual acquires the social knowledge and skills necessary to assume an organizational role’ (Van Maanen & Schein, 1979, p. 211). Successful socialization occurring early in the organizational entry affects the long‐term adjustment outcomes of the employee through, for example, role conflict, role ambiguity, job satisfaction and turnover intent (Ashforth, Sluss, & Harrison, 2007; Saks, Uggerslev, & Fassina, 2007). Van Maanen and Schein's (1979) theory of organizational socialization states that new employees are vulnerable to the influence of individuals that immediately surround them: colleagues, supervisors and the socializing agents, such as preceptors. These social aspects are crucial in facilitating the socialization of new employees because they provide necessary social cues and assistance throughout the learning process (Saks et al., 2007). Thus, preceptors are well positioned to influence how new nurses are socialized during a preceptorship programme. In addition, the literature suggests that the relationship between preceptors and new graduate nurses may be influenced by generational differences, as there may be a clash between differing work values, potentially leading to conflict and differing preceptorship needs (Foley et al. 2012; Keepnews, Brewer, Kovner, & Shin, 2010).

**Figure 1:**
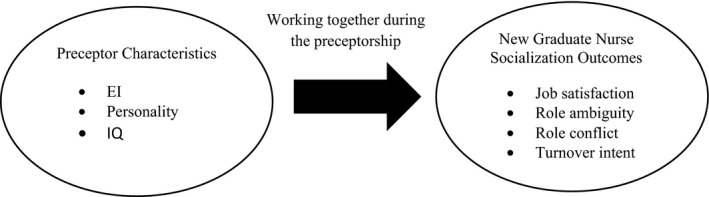
Conceptual model

The literature and Van Maanen and Schein's (1979) theory suggests there is something about an individual preceptor that is important to the socialization process of new nurses. For example Anderson (1998) reported that matching the learning style of new graduates (*n*=26) to those of the preceptor's (*n*=25) teaching style was related to the preceptorship and job satisfaction of new nurses. More recently, Giallonardo, Wong, and Iwasiw (2010) reported that new nurses working with preceptors with higher perceived levels of authentic leadership were more satisfied and engaged with their jobs. These results suggest that preceptor characteristics may have an impact on new nurses and, consequently, decisions about preceptor selection should not be arbitrary. However, there remains a lack of understanding surrounding the links between which specific preceptor characteristics, if any, would be most beneficial to facilitate transition, as well as how these characteristics may influence the transition of new employees (Ashforth et al., 2007; Canadian Nurses Association, 2004, Van Maanen & Schein, 1979). This is one of the limitations of Van Maanen and Schein's theory. However, this paper proposes that preceptor characteristics may influence the socialization outcome of new nurses due to their closeness as teacher and learner during the preceptorship programme (Fig. 1).

Salovey and Mayer (1990) define EI as ‘the ability to monitor one's own and others’ emotions, to discriminate among them and use the information to guide one's thinking and actions’ (p. 189). Mayer et al. (2004) conceptualize EI as comprising four abilities: (a) perceiving emotions; (b) using emotions to facilitate thought; (c) understanding emotions; and (d) managing emotions. There are few studies exploring the relationship between teacher EI and teaching outcomes. The literature reports significant relationships between teacher EI and teaching self‐efficacy (Di Fabio & Palazzeschi, 2008; Mouton, Hansenne, Delcour, & Cloes, 2013; Penrose, Perry, & Ball, 2007). In nursing education, Allen, Ploeg, and Kaasalaine (2012) have examined the relationship between the EI of undergraduate nursing faculty members employed in clinical teaching and teacher effectiveness. The nursing faculty (*n*=47) completed a self‐reported measure of EI, as well as a modified Nursing Clinical Teacher Effectiveness Inventory (NCTEI) on self‐reported teaching effectiveness. The results indicate a positive relationship between faculty EI and perceived teaching effectiveness. These studies on self‐reported EI and teacher effectiveness did not, however, measure student outcomes.

The business and management literature suggests that a leader's EI may have a positive impact on the work‐related outcomes of subordinates, such as performance and job satisfaction (Sy et al., 2006; Wong & Law, 2002). Although preceptors are not leaders, they are in a position of authority throughout the preceptorship experience and are responsible for guiding and evaluating the new nurse's progress and practice. Another important element of the preceptor role is the relationship he or she develops with the new graduate nurse; evidence suggests that EI has a positive influence on interpersonal relationships (Lopes et al., 2003; Quoidbach & Hansenne, 2009). Personality is conceptualized by McCrae and Costa (1997) as ‘variations in basic human ways of acting and experiencing’ (p. 509). The big five personality traits examined include extraversion, agreeableness, conscientiousness, openness and emotional stability. Significant positive relationships and shared variance have been reported between EI, IQ and several personality traits, such as openness, agreeableness and conscientiousness (Mayer et al., 2004; Schulte et al., 2004). This suggests that personality and IQ are important confounding variables in the study of EI. These variables may limit the hypothesis that the individual's EI impacts his or her work outcomes, not their higher intelligence, friendliness and openness to new experiences. As well, the addition of preceptor personality and IQ may enrich our understanding of the potential influence of the preceptor's individual characteristics on the socialization outcomes of new graduate nurses.

Overall, there is a lack of understanding surrounding the impact of preceptor characteristics on new graduate nurses in the context of a preceptorship programme. Additionally, evidence from the literature suggests that the differences between millennial and other generations may impact the relationship between the preceptor and the new graduate nurse. To date, no studies have examined the relationship between preceptor/teacher EI, personality IQ and the socialization outcomes of new graduate nurses/learners. However, the literature indicates that preceptor EI may be an important characteristic to consider. Preceptors with strong emotional abilities may be in a better position to help new graduates navigate the challenges faced during early experiences while facilitating their transition. The purpose of this study was to explore the relationships between preceptor EI, personality and IQ and the socialization outcomes of new graduate nurses, including job satisfaction, role conflict, role ambiguity and turnover intent.

## 2 The study

### 2.1 Design and sample

This study used a cross‐sectional design with purposeful sampling comprised of dyads of new graduate nurses and their preceptors at five large hospitals in the Greater Toronto Area, in Canada, between 2012–2013. Each site participated in the provincial Ontario New Graduate Nurse initiative, thus guaranteeing a minimum of 12 weeks of orientation for new nurses, as well as a contract for 6 months of full‐time employment. Inclusion criteria for the dyads were: (1) must be a new graduate nurse currently in his or her first nursing job; (2) must be within a month of finishing the preceptorship programme; (3) the main preceptor must have been identified; and (4) must have the ability to read and write English. The time criterion was chosen for several reasons. First, the researchers were interested in exploring the transition period of new graduate nurses at the end of a preceptorship programme, at a time in their practice when they were no longer working closely with a preceptor. Additionally, this timeline fit the support period of the Ontario New Graduate Nurse initiative; the new graduates in this study were provided with a preceptorship programme of comparable length. Also, the socialization that takes place during and after a preceptorship programme may be different. Including only those within a month of completing the preceptorship programme may limit the capturing of this potentially different type of socialization.

### 2.2 Participant recruitment and data collection

Each hospital appointed a site contact, usually the Director of Research, or designate, a designated representative in charge of identifying and recruiting eligible participants. The site contacts contacted the unit managers and educators to determine the number of new graduate nurses to be hired, as well as the email addresses of each new graduate and their preceptor. The site contacts and then sent an email containing a description study to the new graduates and preceptors 4, 2 and 1 week prior to the end of the preceptorship programme. This letter described the study and invited interested individuals to contact the researcher. Additionally, the researcher was present at shift changes to introduce nurses to the study and determine if there were new graduates and preceptors working on the unit that day and if they might be interested in receiving further information about the study. New graduate nurses and their preceptors were approached as dyads. Data were collected through the use of a questionnaire completed by participants at the end of the preceptorship programme between 2012–2013.

#### 2.2.1 Measures

Demographic variables included age, gender, years of nursing experience, current unit of employment and highest level of education. Preceptor EI was measured using the Nursing Emotional Intelligence Scale (NEIS), a measure the researcher adapted from the Consumer Emotional Intelligence Scale (CEIS; Kidwell, Hardesry, & Childers, 2008) and pilot tested in a sample of 107 nurses with preceptor experience (Lalonde, 2013). Psychometric testing in the pilot study consisted of assessing the tool's face, discriminant and concurrent validity, as well as its internal structure through factor analysis (Nunnally, 1978). Corresponding to Salovey and Mayer's (1990) ability model, the NEIS was moderately distinct from the Big Five personality traits and had a moderate correlation with another measure of EI, the CEIS. The NEIS consisted of 15 items, four of which were photos asking participants to choose the option that best corresponds to the emotion expressed in the pictures and faces. The remaining 11 questions involved nursing situations where respondents were asked to choose the option that best represents the emotion expressed in the scenario. The NEIS was scored using a scoring key developed from the expert nurses (*n*=16) in the pilot study using the same methodology as the CEIS (Kidwell et al.). Exploratory factor analysis yielded a two‐factor model that explained 36.3% of the variance with a split‐half reliability of 0.70. In this study, the split‐half reliability was 0.62. To ease interpretability, the NEIS was normalized to standard scores with a mean of 100 and a standard deviation of 15.

The Cattell Culture Fair Intelligence Test, Scale 3, Form A, was used to measure preceptor IQ. This involves a timed 50‐item instrument comprised of diagrams that require the participants to solve problems (Hogrefe 2008). Reliability was 0.78. This variable was included due to a reported small to moderate relationship with EI (Schulte et al., 2004).

Four preceptor personality traits were measured using Goldberg's (1999) International Personality Item Pool (IPIP) short scale: (1) agreeableness‐friendly and cooperative; (2) conscientiousness‐reliable, hardworking and thorough; (3) openness to experience‐curious and open to different ways of thinking; and (4) emotional stability‐self‐control and able to remain calm in stressful situations (Hirschfeld, Jordan, Thomas, & Feild, 2008). This tool is comprised of 10 items for each of the four Big Five personality traits on a 5‐point Likert scale ranging from *very inaccurate* to *very accurate*. The alpha coefficients for the IPIP subscales are: α=0.87 for agreeableness, α=0.80 for openness, α=0.84 for conscientiousness and α=0.87 for emotional stability. As previous research has demonstrated a small to moderate correlation between EI and the personality traits of openness, conscientiousness and agreeableness (between r*=*0.1 and 0.3), these were included in this study (Mayer et al., 2004).

Role ambiguity is defined as an employee's lack of clarity and understanding of the expectations and responsibilities surrounding their job (Rizzo, House, & Lirtzman, 1970). The role ambiguity of new graduate nurses was measured using Rizzo et al.'s 6‐item tool on a 7‐point scale ranging from ‘Very false’–‘Very true’. A global score was obtained by summing the items. This measure has been used in previous nursing studies in Ontario with a reported reliability of 0.74 (McGillis Hall, 2003). For this study, this tool received a reliability estimate of α*=*0.83.

Role conflict is defined as an employee's struggle against incongruent and/or incompatible job‐related demands that impact their ability to perform their role (Rizzo et al., 1970). The role conflict of new graduate nurses was measured using Rizzo et al.'s 8‐item tool on a 7‐point scale ranging from ‘Very false’–‘Very true’. A global score was achieved by summing the items, with higher scores representing higher levels of role conflict. The reported reliability of this instrument in a sample of acute care nurses in Ontario was 0.74 (McGillis Hall, 2003). In this study, the reliability estimate was α=0.85.

The job satisfaction of new nurses was measured using the Michigan Organizational Assessment Questionnaire Job Satisfaction Subscale, or MOAQ‐JSS (Cammann, Fichman, Jenkins, & Klesh, 1983). The MOAQ‐JSS asks respondents to answer three questions on a 7‐point Likert scale ranging from ‘Strongly disagree’–’Strongly agree’. Peterson, McGillis Hall, O'Brien‐Pallas, and Cockerill (2011) reported a reliability of 0.88 in a sample of new graduate nurses in Ontario. For this study, the reliability estimate was α=0.93.

The turnover intent of new graduates, or the intention to quit, comprises three areas: thinking of quitting, intending to leave and searching for new employment (Mobley, Horner, & Hollingsworth, 1978). This variable was measured using Mobley et al.'s (1978) scale, which consists of seven items using a 5‐point Likert scale ranging from ‘Strongly agree’—‘Strongly disagree’, with higher scores representing a greater turnover intent. A global turnover intent score was obtained by averaging the three items. The reliability of this instrument has been reported as 0.86 in a sample of nurses (Castle, 2006). For this study, the reliability estimate was α=0.89.

### 2.3 Ethical considerations

Ethics approval was received by the university's research ethics board and by the five participating hospitals. Written consent was obtained once the participants had read through the information letter and consent form and after the researcher had answered their questions. Nurses were informed that their participation in this study would not impact their job evaluation or their employment. No identifying information was collected and code numbers were used for data entry purposes only.

### 2.4 Data analysis

Data were analysed in SPSS version 21. The level of statistical significance was set at *p*<.05. Analysis including descriptive statistics, such as means, standard deviations and frequency distributions were examined. Scatter plots were also examined to determine the existence of outliers and the data were examined to determine if the variables were normally distributed. Descriptive statistics and Pearson's correlational analysis were used to examine the relationship between the variables.

As the role ambiguity and job satisfaction of new graduate were negatively skewed, they were transformed to conform to normality assumptions prior to undertaking correlational analysis (Tabachnick & Fidell, 2007). Role ambiguity was reflected and log transformed, capturing the log of role clarity, with higher scores representing greater role ambiguity. Job satisfaction was reflected and square rooted, with the variable now representing the square root of job dissatisfaction. The variable of turnover intent of new graduate nurses was positively skewed, demonstrating lower turnover intent for study participants in this study. As suggested by Tabachnick and Fidell, transformations were conducted but failed to create a normal distribution, thus violating the normal distribution assumption of regression. Consequently, the variable was dichotomized (Pallant, 2007). Pearson's correlations were applied to the transformed data (Table 2).

## 3 Results

### 3.1 Sample description

A total of 41 preceptors and 44 new graduate nurses participated in this study, making for a total of 38 dyads with complete data. Over half of the participants worked in a paediatric setting, while the remainder worked in adult acute care hospitals. Respondents represented fields that include surgery (*n*=11, 22%), medicine (*n*=9, 18%) and a combination of both (*n*=8, 16%). New graduate nurses ranged in age from 21 to 29 with a mean age of 24.5 years and averaged 4 months of nursing experience. All held a Bachelor's Degree in nursing. Most were female (*n*=39, 78%) and had been hired as temporary employees (*n*=29, 58%) through the New Graduate Nurse initiative (*n*=44, 88%). The mean length of the preceptorship programmes were 3 months (*SD* 0.13), consistent with the Ontario New Graduate Nurse initiative.

The age of preceptors ranged from 23–53 with a mean of 31.6 years and averaged 7.02 years of nursing experience. Most preceptors were female (*n*=38, 76%), employed full‐time (*n*=37, 74%) and half held a Bachelor's Degree in nursing (*n*=25, 50%). A small number (*n*=7, 14%) were enrolled in a Bachelor's or Master's programme in nursing and 20% (*n*=10) held a non‐nursing Bachelor's Degree.

### 3.2 Preceptors

The EI score of preceptors ranged from 63.6–125.1, with higher scores representing greater EI (Table 1). Forty per cent (*n*=16) of preceptor scores fell below the mean, while 60% (*n*=24) rose above. The scores for the personality trait of agreeableness ranged between 1.80 and 5.00, with a mean of 4.36 (*SD* 0.57). The scores for conscientiousness ranged between 2.30–5.00, with a mean of 4.10 (*SD* 0.55). These results suggest these two personality traits were prominent for preceptors in this study. The means for the other two personality traits, openness (3.63, *SD* 0.50, range 2.00–4.70) and emotional stability (3.35, *SD* 0.65, range 2.00–4.70), were just below the midpoint, suggesting the preceptors in this study displayed below‐average scores for these traits. The mean IQ score was 24.6 (*SD* 3.61), with scores ranging from 11–34. Higher scores represent greater IQ and as the median and mode were 24, this suggests little variation among the scores. To aid in the interpretation of the results, IQ scores were normalized as outlined by the instrument developer (Hogrefe 2008). The normalized standard score was 108 at the 69th percentile, suggesting above‐average IQ in this sample of preceptors.

**Table 1:** Preceptor characteristics

Instrument(Scale range)	Median	Mode	Mean (*SD*) (Range)	Number (*N*)
Cattell culture fair intelligence test (Hogrefe, 2008) (Scale range 0–50)	24	24	24.6 (3.61) (11–34)	41
Agreeableness (IPIP, Goldberg, 1999) (Scale range 1–5)	4.40	4.20	4.36 (.57) (1.80–5.00)	40
Conscientiousness (IPIP, Goldberg, 1999) (Scale range 1–5)	4.15	3.70^a^	4.10 (.55) (2.30–5.00)	40
Openness (IPIP, Goldberg, 1999) (Scale range 1–5)	3.70	3.30^a^	3.63 (.50) (2.00–4.70)	40
Emotional Stability (IPIP, Goldberg, 1999) (Scale range 1–5)	3.40	3.60	3.35 (.65) (2.00–4.70)	39
Nursing Emotional Intelligence Scale (NEIS)	104.6	110.5	100 (15) (63.6–125.1)	40

^a^Multiple modes; the smallest value is shown.

### 3.3 New graduate nurse outcome scores

The role ambiguity of new graduate nurses ranged between 2.67–7.00. The mean was 5.75 (*SD* 0.84), which is above the midpoint, indicating that respondents appear to have a good understanding of their nursing role. The role conflict scores ranged from 1.00–6.25, with an average of 3.21 (*SD* 1.17), which is slightly below the midpoint, suggesting that this sample of new graduates experienced low levels of role conflict. The new graduate nurses had little intent to turnover, with a mean of 1.70 (*SD* 0.83) and a range between 1–4. Additionally, they reported high levels of job satisfaction 6.36 (*SD* 0.99), with scores ranging from 1.00–7.00.

### 3.4 Preceptor characteristics and new graduate nurse outcomes

As outlined in Table 2, preceptor EI and IQ were not significantly related to any of the new graduate nurse outcome variables. However, preceptor conscientiousness was moderately correlated with new graduate turnover intent (*r=*0.37, *p=*0.025). Additionally, preceptor openness was moderately correlated with new graduate job dissatisfaction (*r=*0.35, *p=*0.035) and role conflict (*r=*0.40, *p=*0.017). Finally, preceptor emotional stability was positively related to new graduate role clarity (*r=*0.34, *p=*0.039).

**Table 2:** Reliabilities and pearson's correlations for preceptor and new graduate nurse variables

				Preceptor measures						NGN measures			
				1.	2.	3.	4.	5.	6.	7.	8.	9.	10.
Preceptor measures	1.	NEIS	(*n*)	(.62) (40)									
	2.	Agreeableness	*rp* (*n*)	−.04.99 (40)	(.87) (40)								
	3.	Conscientiousness	*rp* (*n*)	.28.15 (40)	.65**.00 (40)	(.84) (40)							
	4.	Openness	*rp* (*n*)	.23.13 (40)	.44**.00 (40)	.39*.01 (40)	(.80) (40)						
	5.	Emotional Stability	*rp* (*n*)	−.18.37 (39)	.38*.02 (39)	.03.86 (39)	.04.84 (39)	(.87) (39)					
	6.	IQ	*rp* (*n*)	−.27.06 (38)	−.11.50 (38)	−.16.35 (38)	−.07.67 (38)	−.21.20 (37)	(.78) (41)				
NGN measures	7.	Job dissatisfaction^a^	*rp* (*n*)	.27.09 (38)	.09.61 (38)	.02.89 (38)	.37*.04 (38)	.02.91 (37)	−.22.18 (37)	(.93) (44)			
	8.	Turnover intent^b^	*rp* (*n*)	.10.71 (37)	.31.07 (37)	.37*.03 (37)	.21.22 (37)	.02.90 (36)	−.14.40 (37)	.24.12 (43)	(.89) (44)		
	9.	Role conflict	*rp* (*n*)	.31.05 (35)	.22.21 (35)	.12.51 (35)	.40*.02 (35)	.29.09 (34)	.02.89 (35)	.72**.00 (41)	.10.51 (42)	(.85) (42)	
	10.	Role ambiguity^c^	*rp* (*n*)	.07.41 (38)	.24.14 (38)	.11.51 (38)	.15.37 (38)	.34*.04 (37)	−.29.08 (38)	.43*.03 (43)	−.00.99 (43)	.32*.04 (41)	(.83) (44)

*p<.05; **p<.01.

^a^Variable reflected and square root transformed : higher scores represent higher square root of job dissatisfaction.

^b^Variable transformation‐Dichotomized: 0 = low turnover intent and 1 = high turnover intent.

^c^Variable reflected and log transformed : higher scores represent higher role ambiguity.

## 4 Discussion

Three preceptor personality traits were related to new graduate nurse outcomes. First, preceptor openness was positively correlated with new graduate job dissatisfaction and role conflict. This indicates that the more open the preceptor, the higher the job dissatisfaction among new graduate and the higher their perception of role conflict. This cohort of new graduate nurses, with an average age of 24, is considered part of Generation Y, or the millennial generation, that is, individuals born between 1979 and 1994 (Myers & Sadaghiani, 2010). Millennials are considered to differ on important workplace characteristics compared with other generations, such as those involving work–life balance and organizational commitment (Myers & Sadaghiani, 2010). Keepnews et al. (2010) reported that the new graduate nurse millennials in their study had higher negative moods compared with the other generations. Myers and Sadaghiani (2010) suggest that conflict can occur when there are differences between the work‐related values and role expectations of colleagues from different generations. Foley, Myrick, and Yonge (2012) report that the intergenerational conflict found in their study of nurses involved in nursing preceptorships may have been due to a lack of generational knowledge.

Second, preceptor conscientiousness was positively related to new graduate turnover intent. Specifically, the more conscientious the preceptor, the higher the intent to turnover among the new graduate participants. This implies that new nurses paired with more conscientious preceptors are more likely to want to leave their current job. As the preceptors provide the technical information surrounding their new role, along with important social cues, the new nurse assesses these social cues and the people surrounding them (Saks et al., 2007; Van Maanen & Schein,^1979^). As such, perhaps more conscientious preceptors have higher expectations regarding the new nurse during his or her early work experience and these may influence the new nurse's intent to turnover. Myers and Sadaghiani (2010) suggest that millennials have greater work–life balance expectations than previous generations.

Third, preceptor emotional stability was positively related to new graduate role ambiguity, suggesting that new nurses paired with preceptors who were calmer and less reactive to stress were more likely to experience role ambiguity. This finding was unexpected and additional research is required to further explore these relationships. It may provide important information about the realities of current acute care hospital work environments and this particular cohort of new nurses.

The lack of significant findings between preceptor EI, IQ and new graduate work outcomes in this sample suggests that EI and IQ did not impact new graduate outcomes. It is possible that preceptor EI is not as important a contributor to new graduate nurse outcomes than specific personality traits. Perhaps the continued support provided by the preceptor during the extended preceptorship programme was the most important element for the new graduates during their transition period. Peterson et al. (2011) explored factors that contributed to the job satisfaction and turnover intent of Canadian new graduate nurses (*n*=232). These authors report that social support from both supervisors and coworkers were significantly related to new graduate job satisfaction, while support from coworkers was related to new nurse turnover intent. Therefore, the support provided by the preceptor may be a significant component.

The majority of preceptors had EI scores that rose above the mean, suggesting a higher EI. This finding is similar to what has been reported in a sample of North American undergraduate faculty members employed in clinical teaching (Allen et al., 2012). By contrast, Saeed, Javadi, and Nouri (2013) reported that, in their sample of Iranian nurses (*N*=212), 48.6% (*N*=103) were categorized as having ‘good’ or ‘excellent’ EI skills, whereas 51.5% (*N*=109) fell in the low EI categories. One third of the sample in this study fell below the mean, suggesting lower EI. These findings are similar to those reported in US staff nurses by Codier, Kooker, and Shoultz (2008). The low EI found in more than a third of the study's preceptor participants is a cause for concern given the nature of nursing work. Although some nurses opt to be preceptors, the literature indicates that preceptors are often selected by nursing managers, regardless of their interest in the role. Preceptors in this study were not asked if they had volunteered for the role and therefore it is not possible to determine if there is a difference in EI scores between nurses who volunteer for the role and those who do not.

Preceptor IQ was not significantly related to any new nurse outcome. The IQ results suggest above‐average IQs with little variation in scores. These findings are interesting and may be explained by the specific types of individuals who are attracted to the nursing profession and by the nature of the nurses’ educational preparation. Half of this preceptor sample held Bachelor's Degrees and a quarter held Graduate Degrees. In 2005, an Ontario Bachelor's Degree in nursing became the entry‐to‐practice requirement and all nurses were required to write the same entry‐to‐practice registration exam, which sets the minimum provincial standard. It is possible that the dynamic and complex profession of nursing attracts individuals who tend to have higher IQs. One paradoxical finding involves a lack of variation in preceptor IQ scores, while the EI scores do vary, with a low EI in one third of the participants. This warrants further investigation. More specifically, the relationship between EI and IQ in nurses as well as the role of IQ in the nursing profession are not yet known.

### 4.1 Implications for Research and Practice

The results of this study provide preliminary support to the idea that preceptor characteristics may influence the outcomes regarding new graduate nurses. More specifically, the influence of preceptor personality traits should be investigated further. Future research should examine whether or not personality differences exist between nurses who volunteer to be preceptors and those who do not and whether or not personality traits are related to new graduate satisfaction surrounding both preceptorship programmes and outcomes. Further research should also examine what combinations of preceptor/new graduate personality traits lead to optimal new graduate outcomes. Additionally, future research should examine the impact of the differences between millennials and previous generations to ensure that current preceptorship programmes meet millennial needs, while further consideration is needed to determine how these generational differences may impact the preceptor–preceptee relationship and work environment.

The literature suggests that EI could have an impact on several important preceptorship related elements, such as performance, teaching self‐efficacy and interpersonal relationships. In terms of the effects of EI on teaching, very few studies have been conducted. Among those, none have explored the relationship between EI, personality, IQ and teacher effectiveness, along with their possible link with students learning outcomes. In addition, no studies have yet examined the impact of personality on preceptorship outcomes. Further research in nursing is needed to deepen our understanding of the impact that EI and personality may have on nursing practices and teaching in a clinical setting.

Finally, the relationship between individual preceptor characteristics and new graduate nurse socialization outcomes may not be as simple as originally proposed in this study. Preceptors are undoubtedly important to this process, but the How remains unclear. Future studies examining the impact of preceptors on new graduate outcomes should consider exploring the relationship between new nurses and their preceptors. The quality of the relationship may also impact new nurse socialization. It would be worthwhile to consider certain elements of this dyad relationship, such as the relationship's development over time, trust, the supportiveness of the preceptor, the quality of the relationship, as well as the support provided by the preceptor during extended preceptorship programmes. Van Maanen and Schein's (1979) theory of organizational socialization provides a strong theory for understanding the process of socialization and acknowledges the importance of the socializing agent in this process. However, the theory contains gaps. Primarily, it does not explore how the socializing agent specifically impacts the new employee, or which characteristics are important in the process.

Preceptors and new graduate nurses do not work and learn in a context that is isolated from other factors involved in the socialization process. The context where nurses work should be included in any further research on this topic. Future studies should also consider the impact of environmental factors on the socialization of new nurses, such as staffing, skill mix, unit culture and leadership style, along with patient acuity and needs.

### 4.2 Limitations

The number of available participant dyads for this study was less than expected, which limits the generalizability of results. Preceptor EI was measured using the NEIS, a scale adapted for this study and pilot‐tested prior to use. The results of the pilot test suggest that the NEIS was reliable and valid. However, the reliability of the pilot dropped from 0.70–0.63. Further research is needed to examine the validity and reliability of the NEIS in different samples of nurses. To date, there are no other tools that specifically measure the EI of nurses. Therefore, a valid and reliable measure of nurse EI would be a significant contribution to the field. The impact of important environmental variables on the socialization of new nurses, such as staffing, skill mix, patient acuity and needs, fell beyond the scope of this study and thus limits the generalizability of results. The context where nurses work and teach should be included in any future research on this topic.

## 5 Conclusion

This study examined the impact of preceptor characteristics, EI, IQ and personality on the socialization outcomes of new graduate nurses. Three preceptor personality traits, including openness, conscientiousness and emotional stability, were related to new graduate nurse outcomes. This study provides additional support to the existing literature that examines how preceptor characteristics may be worthy of consideration. However, further research is required to determine whether or not preceptor personality traits impact the socialization outcomes of new graduate nurses.

## Funding

The authors acknowledge the following sources of funding: Lawrence S. Bloomberg Faculty of Nursing Rosenstadt Doctoral Research Dissertation Award, University of Toronto; Registered Nurses Foundation of Ontario; and CHSRF/CIHR Ontario Training Center in Health Services and Policy Research.

## Conflict of interest

No conflict of interest has been declared by the author(s).

## Author Contribution

All authors have agreed on the final version and meet at least one of the following criteria [recommended by the ICMJE (http://www.icmje.org/recommendations/)]:

- substantial contributions to conception and design, acquisition of data, or analysis and interpretation of data;
- drafting the article or revising it critically for important intellectual content.
